# Prevalence and factors associated with self-reported diagnosis of high cholesterol in the Brazilian adult population: National Health Survey 2019

**DOI:** 10.1590/SS2237-9622202200002.especial

**Published:** 2022-06-29

**Authors:** Ana Carolina Micheletti Gomide Nogueira de Sá, Crizian Saar Gomes, Alexandra Dias Moreira, Gustavo Velasquez-Melendez, Deborah Carvalho Malta

**Affiliations:** 1 Universidade Federal de Minas Gerais, Programa de Pós-Graduação em Enfermagem, Belo Horizonte, MG, Brazil; 2 Universidade Federal de Minas Gerais, Faculdade de Medicina, Belo Horizonte, MG, Brazil; 3 Universidade Federal de Minas Gerais, Escola de Enfermagem, Belo Horizonte, MG, Brazil

**Keywords:** Dyslipidemia, Hypercholesterolemia, Cholesterol, Health Surveys, Risk Factors, Cross-Sectional Studies

## Abstract

**Objective:**

To estimate the prevalence of self-reported high cholesterol diagnosis and to analyze the factors associated with the prevalence in the Brazilian adult population.

**Methods:**

Cross-sectional study, using data from the 2019 National Health Survey. The diagnosis of high cholesterol was self-reported. Poisson regression models yielded prevalence ratios (PR) and 95% confidence intervals (95%CI).

**Results:**

In the 88,531 adults, the prevalence of high cholesterol was 14.6%. Positively associated: female sex (PR = 1.44; 95%CI 1.40;1.52), age ≥ 60 years (PR = 3.80; 95%CI 3.06;4.71), health insurance (PR = 1.33; 95%CI 1.24;1.42), poor or very poor self-rated health (PR = 1.75; 95%CI 1.60;1.90), hypertension (PR = 1.78; 95%CI 1.68;-1.89), diabetes (RP = 1.54; 95%CI 1.45;1.65), renal failure (PR = 1.33; 95%CI 1.15;1.53), obesity (PR = 1.27; 95%CI 1.18;1.36), former smoker (PR = 1.13; 95%CI 1.07;1.20), alcohol abuse (PR = 1.11; 95%CI 1.01;1.21), physically active during leisure time (PR = 1.22; 95%CI 1.15;1.30).

**Conclusion:**

High cholesterol was associated with sociodemographic characteristics, health condition and lifestyle.

**Study contributions**

**Main results**

In Brazil, 14.6% of adults reported having high cholesterol, and were factors associated being sex female, ageing, socioeconomic status, worst self-assessment of health, chronic diseases, overweight/obesity, race/ Black/Brown skin color, behavioral habits and lifestyles.

**Implications for services**

The results of this study can provide support for health promotion public policies, for the development of clinical protocols within the scope of the Brazilian National Health System (SUS), and for supporting actions related to the prevention and reduction of dyslipidemia and cardiovascular diseases.

**Perspectives**

The high prevalence of self-reported high cholesterol in the Brazilian adult population, identified in the 2019 National Health Survey, implies that monitoring dyslipidemia is crucial for the prevention of cardiovascular diseases in the country.

## Introdution

Dyslipidemia is characterized by abnormal concentrations of lipid circulating in the bloodstream, such as total cholesterol, triglycerides, low density lipoprotein (LDL), or high-density lipoprotein (HDL).^1^

Elevations in the levels of total cholesterol, triglycerides, and mainly LDL, increase the risks of cardiovascular and cerebrovascular diseases.^2^ According to the last report on high levels of serum cholesterol issued by the World Health Organization (WHO), published in 2009, it caused 2.6 million deaths (4.5% of the total) and 29.7 million disability adjusted life years (DALYs) throughout the world.^3^ In middle-income countries it was responsible for 1.3 million deaths (5.2% of the total), and 14 million DALYs (2.5% of the total).^3^ Worldwide, high levels of LDL caused, in 2019, 4,396,983 deaths (7.8% of the total) and 98,618,020 DALYs (3.9% of the total)^4^ and, in Brazil, 99,375 deaths (7.0% of the total) and 2,363,140 DALYs (3.6% of the total).^4^

Globally, adult populations are exposed to illnesses and health problems, due to high levels of total cholesterol and fractions,^5^ as a consequence of unhealthy lifestyles, chronic diseases or genetic factors.^1^

Regarding the factors associated to dyslipidemia, the literature documents sociodemographic characteristics,^5^ ^-^ ^8^ inadequate lifestyles,^6^ ^-^ ^8^ altered body mass index (BMI),^5^ ^,^ ^7^ ^,^ ^8^ ^,^ ^9^ noncommunicable chronic diseases (NCDs), ^5^ ^,^ ^7^ ^-^ ^9^ and poor self-rated health status.^7^

Considering the negative impacts of dyslipidemia on cardiovascular health, this study takes a step forward by identifying, for the first time, the prevalence of self-reported diagnosis of high cholesterol and its associated factors in the adult population, using data from the National Health Survey (*Pesquisa Nacional de Saúde* - PNS), 2019 edition. The last PNS that collected self-reported data was conducted in 2013^7^ ^,^ ^10^ and the prevalence of high cholesterol in the Brazilian adult population was 12.5%.^10^ Furthermore, in view of the higher prevalence of dyslipidemia due to alterations in the lipid profile according to the 2014 and 2015 PNS’s laboratory tests database (32.7% high total cholesterol,^11^ 18.6% high LDL^5^ ^,^ ^11^ and 31.8% low HDL^5^ ^,^ ^11^, it is important to understand the present scenario of this condition in the country. The results can contribute to supporting public policies and control measures and, as a result, to the prevention of dyslipidemia.

Thus, the objective of this study was to estimate the prevalence of self-reported high cholesterol diagnosis and to analyze the factors associated with its prevalence in the Brazilian adult population.

## Methods

### Study design

This was a cross-sectional study that used data from the 2019 PNS, which was conducted between August 2019 and March 2020.

### Context

The PNS is a national-level, population-based household health survey conducted by the Brazilian Institute of Geography and Statistics (IBGE), in partnership with the Ministry of Health. The PNS uses a probability sample design and three-stage stratification: censor tracts, households and residents,^12^ with the last two being selected by simple random sampling.^12^ ^,^ ^13^ In the 2019 PNS edition, in the third stage of selection, one resident from each household was randomly selected among those aged 15 years and older, based on a list of household residents obtained at the time of the interview.^12^ ^,^ ^13^

However, the following were excluded from the PNS: barracks, military bases, prisons, indigenous communities, lodgings, campsites, farming settlements, quilombola communities, waterborne vessels, convents and monasteries, hospitals and long-stay institutions for the elderly, children or adolescents. Further details on the methodological procedures are described in previous publications.^12^ ^,^ ^13^

Due to PNS’s complex sampling design, sample weighting for households and selected residents were defined. The final weighting is the product of the inverse of the selection probabilities at each stage of the sampling plan, aiming to correct losses and to make adjustments to the population totals.^12^ ^,^ ^13^

### Participants

For the present study, data from selected residents^12^ aged ≥ 18 years were used.

### Data source

The 2019 PNS data which were used and its database and questionnaires are open access and were obtained through the PNS repository, available from https://www.pns.icict.fiocruz.br/.

The PNS questionnaire is divided in modules, containing information about place of residence, all the residents, and the resident selected for the interview.^12^ ^,^ ^13^ In this study, questions from the following modules were used: Identification; Residents’ characteristics (C); Level of education (D); Health insurance coverage (I); Self-perceived health (N); Lifestyle (P); and Chronic diseases (Q).^12^ ^,^ ^13^

### Variables

The outcome variable was the self-reported diagnosis of high cholesterol, evaluated by the following question: *Has any doctor ever told you that you have high cholesterol?* A diagnosis of high cholesterol was considered the answer “yes”.

Explanatory variables included:

- *Sociodemographic characteristics:* sex (male; female); age group in years (18 to 24; 25 to 39; 40 to 59; ≥ 60); level of education (no schooling and incomplete primary education; complete primary education and incomplete secondary education; complete secondary education and incomplete higher education; complete higher education); race/skin color [White; Brown; Black; and others (corresponding to Yellow and Indigenous)]; region (North; Northeast; Southeast; South; Midwest); health insurance coverage (yes; no) - based on the questions on the identification module and the C, D, and I modules, and the questionnaire.
- *Self-perceived health:* self-rated health (very good/good; fair; poor/very poor); self-reported diagnosis of hypertension (yes; no); self-reported diagnosis of diabetes (yes; no); self-reported diagnosis of renal failure (yes; no); nutritional status (low weigth/eutrophic, classified by the body mass index [BMI] < 25 kg/m^2^); overweight (BMI around 25 to 29 kg/m^2^); obesity (BMI ≥ 30kg/m^2^).^14^
- *Lifestyle:* smoking (non-smoker; former smoker; smoker); abusive consumption of alcoholic beverages (yes - consumption of five or more drinks on a single occasion; no);^13^ recommended consumption of fruits and vegetables (yes - consumption of such foods at least 25 times a week, with a minimum consumption of five fruits, including fruit juice, or five vegetables; no);^15^ consumption of ultra-processed foods (yes - reported consumption of five or more groups of ultra-processed foods on the previous day; no);^13^ sufficient leisure time physical activity (yes - the following was considered as active: spending 150 minutes per week on activities of light or moderate intensity, or 75 minutes a week on vigorous-intensity activities, regardless of the number of days; no).^16^

For the individuals who reported a diagnosis of high cholesterol, the following aspects were also investigated: age at first diagnosis of high cholesterol (mean age of first diagnosis) and recommendations from the health professional regarding high cholesterol: keeping a healthy diet (yes; no); maintaining adequate weight (yes; no); maintaining regular physical activity (yes; no); use of medication (yes; no); not smoking (yes; no); regular follow-ups with a health professional (yes; no).

Further details on the construction of variables for this study and the calculation methods are presented in the Supplementary material 1.

### Statistical analysis

In the descriptive analyses, prevalences were estimated, presented as proportions (%) and with 95% confidence intervals (95%CI). The completeness of the variables was also analyzed descriptively to identify incomplete data (completeness was above 99% for all variables).

To verify the associations between the outcome and the explanatory variables, the prevalence ratio (PR) was used as a measure of association, obtained by Poisson regression models with robust variance. The theoretical model of the study by Sá et al.^5^ was considered. Bivariate analyzes were performed to obtain the crude PRs (cPR) and 95%CI. A multivariable analysis was performed, including variables with p-value < 0.20 in the crude analyses, to calculate the adjusted PRs (aPR) and 95%CI. The forward method was used to select the variables. In the final model, variables with p-value < 0.05 were considered as associated factors. Confounding variables were tested considering aspects in the literature.^5^ ^-^ ^9^

The analyses were performed by means of the Software for Statistics and Data Science (Stata), version 14, using the survey module for complex samples, which incorporates post-stratification weights.

### Ethical aspects

The 2019 PNS followed all the ethical principles for the conduct of research involving human subjects and was approved by the National Research Ethics Committee of the Ministry of Health, Opinion No. 3,529,376. All participants who were interviewed provided free and informed consent and agreed to participate in the study; confidentiality of their information was guaranteed.

## Results

The 2019 PNS anticipated sample was 108,525 households and the final sample consists of 94,114 households. For the analyses in this study, the total is 88,531 individuals age ≥ 18 years.

The prevalence of self-reported medical diagnosis of high cholesterol was 14.6% (95%CI 14.1;15.0). The mean age at the time of first medical diagnosis of high cholesterol was 45.9 years (95%CI 45.4;46.4; standard deviation = 16.5). Individuals with high cholesterol reported receiving recommendations from health professionals for maintaining a healthy diet (94.1%) and adequate weight (88.3%); and regular physical activity (87.9%); and for using cholesterol medication (74.2%), not smoking (60.7%), and having regular follow-ups with a health professional (74.0%) (Table 1).

**Table 1:** Characteristics of adult Brazilians with self-reported diagnosis of high cholesterol, 2019 National Health Survey, Brazil

Variables	n	% (95%CI^a^)
Diagnosis of high cholesterol	88,531	
Yes		14.6 (14.1;15.0)
No		85.4 (84.9;85.9)
**Age at first diagnosis of high cholesterol (mean; 95%CI^a^)**	13,396	45.9 (45.4;46.4)
**Recommendations from health professionals due to high cholesterol**		
**Maintaining a healthy diet**	13,396	
Yes		94.1 (93.3;94.8)
No		5.9 (5.2;6.7)
**Maintaining adequate weight**	13,396	
Yes		88.3 (87.1;89.3)
No		11.7 (10.7;12.7)
**Doing regular physical activity**	13,396	
Yes		87.9 (86.9;88.9)
No		12.1 (11.1;13.1)
**Use of medication**	13,396	
Yes		74.2 (72.6;75.6)
No		25.8 (24.4;27.3)
**Not smoking**	13,396	
Yes		60.7 (59.0;62.3)
No		39.3 (37.7;40.9)
**Regular follow-ups with a health professional**	13,396	
Yes		74.0 (72.5;75.5)
No		26.0 (24.6;27.5)

a) 95%CI: 95% confidence interval.

The prevalence of high cholesterol was higher in: females (17.6%; 95%CI 17.0;18.3), the elderly (27.2%; 95%CI 26.2;28.3), inhabitans from the Southeast region (15.8; 95%CI 14.9;16.7), individuals who have health insurance (18.3%; 95%CI 17.4;19.2), those who self-rated their health as poor or very poor (31.3%; 95%CI 29.1;33.6), individuals with hypertension (30.9%; 95%CI 29.9;32.0), diabetes (39.2%; 95%CI 37.2;41.2), renal failure (33.7%; 95%CI 29.3;38.4), and obesity (19.7%; 95%CI 18.7;20.7), former smokers (19.5%; 95%CI 18.6;20.4); and for those who consumed fruits and vegetables as recommended (18.2%; 95%CI 17.0;19.5). On the other hand, the lowest prevalence of high cholesterol was present in individuals with intermediate education (complete elementary school and incomplete secondary school, 11.0%; 95%CI 10.1;12.0), of Black race/skin color (13.0%; 95%CI 11.9;14.2), who consumed alcoholic beverages abusively (10.6%; 95%CI 9.7;11.6), who consumed five or more groups of ultra-processed foods (9.8%; 95%CI 8.9;10.7), and who were physically active during leisure time (14.0%; 95%CI 13.2;14.8) (Table 2).

**Table 2:** Prevalence, crude prevalence ratios and 95% confidence intervals for self-reported diagnosis of high cholesterol among Brazilian adults, according to sociodemographic characteristics, clinical conditions and lifestyle, 2019 National Health Survey, Brazil

Variables	n % (95%CI)	High cholesterol	cPR^a^ (95%CI^b^)
**Total**	88,531	14.6 (14.1;15.0)	
**Sociodemographic characteristics**			
**Sex**	88,531		
Male^c^		11.1 (10.6;11.7)	1.00
Female		17.6 (17.0;18.3)	1.58 (1.49;1.68)
**Age group (years)**	88,531		
18 to 24^c^		3.5 (2.8;4.2)	1.00
25 to 39		6.7 (6.1;7.4)	1.93 (1.5;2.41)
40 to 59		17.7 (16.9;18.5)	5.11 (4.17;6.26)
≥ 60		27.2 (26.2;28.3)	7.85 (6.40;9.62)
**Education**	88,531		
No schooling and incomplete primary education^c^		19.1 (18.4;19.9)	1.00
Complete primary education and incomplete secondary education		11.0 (10.1;12.0)	0.58 (0.5;0.63)
Complete secondary education an incomplete higher education		11.3 (10.6;12.0)	0.59 (0.5;0.64)
Complete higher education		15.1 (14.1;16.2)	0.79 (0.73;0.85)
**Race/skin color**	88,522		
White^c^		16.2 (15.5;16.9)	1.00
Brown		13.4 (12.8;13.9)	0.82 (0.77;0.87)
Black		13.0 (11.9;14.2)	0.80 (0.73;0.89)
Others (Yellow/Indigenous)		16.0 (12.2;20.7)	0.98 (0.75;1.29)
**Region**	88,531		
North^c^		11.8 (11.0;12.6)	1.00
Northeast		14.2 (13.6;14.8)	1.20 (1.11;1.30)
Southeast		15.8 (14.9;16.7)	1.34 (1.22;1.46)
South		14.1 (13.3;15.0)	1.20 (1.10;1.31)
Midwest		13.0 (12.0;14.1)	1.11 (0.99;1.23)
**Health insurance**	88,531		
No^c^		13.2 (12.8;13.7)	1.00
Yes		18.3 (17.4;19.2)	1.38 (1.31;1.46)
**Health conditions**			
**Self-rated health status**	88,531		
Good/very good^c^		10.5 (10.0;11.0)	1.00
Fair		20.6 (19.8;21.5)	1.96 (1.85;2.10)
Poor/very poor		31.3 (29.1;33.6)	2.97 (2.72;3.24)
**Hypertension**	88,531		
No^c^		9.4 (9.0;9.9)	1.00
Yes		30.9 (29.9;32.0)	3.29 (3.12;3.47)
**Diabetes**	88,531		
No^c^		12.5 (12.1;12.9)	1.00
Yes		39.2 (37.2;41.2)	3.13 (2.94;3.33)
**Renal failure**	88,531		
No^c^		14.3 (13.9;14.7)	1.00
Yes		33.7 (29.3;38.4)	2.36 (2.10;2.70)
**Nutritional status**	87,678		
Low weight/eutrophic^c^		10.7 (10.2;11.3)	1.00
Overweight		16.3 (15.6;17.1)	1.53 (1.43;1.62)
Obese		19.7 (18.7;20.7)	1.84 (1.71;1.98)
**Lifestyle**			
**Smoking**	88,531		
Non-smoker^c^		13.1 (12.6;13.7)	1.00
Former smoker		19.5 (18.6;20.4)	1.48 (1.40;1.57)
Smoker		11.2 (10.3;12.3)	0.86 (0.78;0.94)
**Heavy episodic drinking**	88,531		
No^c^		15.4 (14.9;15.9)	1.00
Yes		10.6 (9.7;11.6)	0.69 (0.63;0.80)
**Recommended consumption of fruits and vegetables**	88,531		
No^c^		14.0 (13.6;14.5)	1.00
Yes		18.2 (17.0;19.5)	1.30 (1.21;1.39)
**Consumption of ultra-processed foods > 5**	88,531		
No^c^		15.38 (14.9;15.87)	1.00
Yes		9.8 (8.9;10.7)	0.63 (0.57;0.70)
**Sufficient leisure time physical activity**	88,531		
No^c^		14.8 (14.4;15.3)	1.00
Yes		14.0 (13.2;14.8)	0.94 (0.90;1.01)

a) cPR: Crude prevalence ratio; b) 95%CI: 95% confidence interval; c) Reference category.

In the final multivariate model, it was found that being female (aPR = 1.44; 95%CI 1.40;1.52), people with advanced age (25 to 39 years: aPR = 1.67; 95%CI 1.33;2.08; 40 to 59 years: aPR = 3.33; 95%CI 2.70;4.11; ≥ 60: aPR = 3.80; 95%CI 3.06;4.71), having health insurance (aPR = 1.33; 95%CI 1.24;1.42), self-rated health as fair (aPR = 1.40; 95%CI 1.32;1.50) and poor or very poor (PR = 1.75; 95%CI 1.60;1.90), having hypertension (PR = 1.78; 95%CI 1.68;1.89), diabetes (aPR = 1.54; 95%CI 1.45;1.65 ) and renal failure (aPR = 1.33; 95%CI 1.15;1.53), being overweight (aPR = 1.26; 95%CI 1.20;1.33), obese (aPR = 1.27; 95%CI 1.18;1.36), a former smoker (aPR = 1.13; 95%CI 1.07;1.20), drinking alcoholic beverages in excess (aPR = 1.11; 95%CI 1.01;1.21), and being physically active during leisure time (aPR = 1.22; 95%CI 1.15;1.30) were associated with higher prevalence of high cholesterol. Having an intermediate level of education (complete elementary school and incomplete secondary school, aPR = 0.89; 95%CI 0.81;0.98), being of Brown race/skin color (aPR = 0.91; 95%CI 0.86;0.97) and Black (aPR = 0.84; 95%CI 0.77;0.93), and a smoker (aPR = 0.88; 95%CI 0.80;0.97) were associated with lower prevalence of high cholesterol (Table 3).

**Table 3:** Prevalence ratios and 95% confidence intervals, from the final multivariable Poisson regression model, for factors associated with self-reported diagnosis of high cholesterol among Brazilian adults (n = 87,669), 2019 National Health Survey, Brazil

Variables	aPR^a^ (95%CI^b^)
Sociodemographic characteristics	
**Sex**	
Male^c^	1.00
Female	1.44 (1.40;1.52)
**Age group (years)**	
18 to 24^c^	1.00
25 to 39	1.67 (1.33;2.08)
40 to 59	3.33 (2.70;4.11)
≥ 60	3.80 (3.06;4.71)
**Education**	
No schooling and incomplete primary education^c^	1.00
Complete primary education and incomplete secondary education	0.89 (0.81;0.98)
Complete secondary education and incomplete higher education	1.02 (0.95;1.10)
Complete higher education	1.04 (0.95;1.14)
**Race/skin color**	
White^c^	1.00
Brown	0.91 (0.86;0.97)
Black	0.84 (0.77;0.93)
Others (Yellow/Indigenous)	0.97 (0.75;1.25)
**Health insurance**	
No^c^	1.00
Yes	1.33 (1.24;1.42)
**Health conditions**	
**Self-rated health status**	
Good/very good^c^	1.00
Fair	1.40 (1.32;1.50)
Poor/very poor	1.75 (1.60;1.90)
**Hypertension**	
No^c^	1.00
Yes	1.78 (1.68;1.89)
**Diabetes**	
No^c^	1.00
Yes	1.54 (1.45;1.65)
**Renal failure**	
No^c^	1.00
Yes	1.33 (1.15;1.53)
**Nutritional status**	
Low weight/eutrophic^c^	1.00
Overweight	1.26 (1.20;1.33)
Obese	1.27 (1.18;1.36)
**Lifestyle**	
**Smoking**	
Non-smoker^c^	1.00
Former smoker	1.13 (1.07;1.20)
Smoker	0.88 (0.80; 0.97)
**Heavy episodic drinking**	
No^c^	1.00
Yes	1.11 (1.01;1.21)
**Physically active during leisure time**	
No^c^	1.00
Yes	1.22 (1.15;1.30)

a) aPR: Adjusted prevalence ratio; b) 95%CI: 95% confidence interval; c) Reference category.

## Discussion

One out of seven adult Brazilians reported a diagnosis of high cholesterol, as per the 2019 PNS data. Prevalence was positively associated with: being female; advanced age; having health insurance; fair, poor or very poor self-rated health; having hypertension, diabetes, renal failure; being overweight or obese; being a former smoker; abusive alcohol consumption; and being physically active during leisure time. They were inversely associated with having completed elementary and secondary education, being of Black and Brown race/skin color, and being a smoker.

Among the limitations of this study are those inherent to cross-sectional studies: the inability to determine causality; the associations presented were analyzed at a single point in time and can be influenced by changes in lifestyle and treatment; some results can be subject to survival bias, such as the difference in terms of associations in relation to sex; and possible reverse causality between NCDs, lifestyle and dyslipidemia variables. Another limitation is the fact that the survey collected self-referred information, being subject to information and diagnosis classification biases, which may lead to underreporting or underestimation.^7^ Even though clinical laboratory measurements are more accurate, population studies on dyslipidemia with laboratory test data are scarce in Brazil due to high costs.^7^ ^,^ ^11^ Studies using self-reported information are important for monitoring the prevalence of high cholesterol,^7^ as they are a faster and more inexpensive way to obtain data.^10^ Furthermore, the study was conducted with a representative sample of the population and the generalizations of estimates are relatively safe.

In adults, the mean age at the time of first diagnosis was approximately 46 years. In the 2013 PNS, the mean age was 46.7 years.^10^ In the present study, there was high prevalence of recommendations from health professionals to adults with high cholesterol in terms of adopting healthy behaviors and health care such as medication use and monitoring. Similar results were found in the 2013 PNS.^10^ Literature establishes the importance of adopting a healthy diet, maintaining an adequate BMI, as well as regular physical activity in order to prevent and control dyslipidemia.^1^ In addition, those individuals also benefit from treatment with hypolipidemic agents, as the reduction in total cholesterol, mainly LDL, decreases morbidity and mortality resulting from cardiovascular diseases.^1^

The prevalence of high cholesterol found in the 2019 PNS was higher than in the 2013 PNS (12.5%; 95%CI 12.1;13.0).^10^ Studies in Brazil,^5^ ^,^ ^10^ ^,^ ^11^ Turkey,^9^ the United States,^17^ and China^18^ identified higher laboratory prevalence of dyslipidemias^5^ ^,^ ^10^ ^,^ ^11^ ^,^ ^17^ ^,^ ^18^ than those found in this study.

The increase of dyslipidemia between the 2013^10^ and 2019 editions of the PNS may be a consequence of greater detection, as a result of improvements and the expansion of access to as well as of use of health services in the country,^19^ but it may also be related to the increase in overweight, obesity and the consumption of ultra-processed foods.^20^ The difference between self-reported and laboratory data may suggest underestimation of diagnosis in the population studied.^21^ In this context, monitoring dyslipidemia in the country^7^ is crucial for the prevention of cardiovascular diseases, which are the main cause of mortality in Brazil.^4^

This study is in line with other investigations that identified a higher prevalence of dyslipidemia in women.^5^ ^,^ ^8^ ^,^ ^10^ ^,^ ^18^ Dyslipidemia is highly prevalent among women,^22^ with higher occurrence with increasing age, during pregnancy, menopause and post-menopause due to hormonal changes.^22^ With advancing age, the increase in levels of triglycerides, total cholesterol, LDL, and reduction of HDL is accentuated.^22^ As people age, changes in the lipid profile place women at greater risk of cardiovascular diseases.^22^ In Brazil, data from the 2013 PNS showed a positive association in women, with or without chronic diseases, when compared to men, regarding greater use of health services and number of doctor visits in the last 12 months,^23^ favoring diagnosis and treatment in this group.

As in other studies, this investigation identified a positive association between age and the diagnosis of high cholesterol.^5^ ^-^ ^7^ ^,^ ^9^ Dyslipidemias are more prevalent with increasing age^24^ due to ageing of the main organs of homeostasis, resulting in changes in the hepatic endothelium, in increased insulin resistance and hormonal changes, such as the decrease in estrogen and progesterone in women and androgen in men throughout life, leading to repercussions on the lipid profile.^24^ Another possible explanation for this finding is the fact that, in Brazil, elderly people use healthcare services more frequently, which contributes to the diagnosis.^19^

There was lower prevalence of high cholesterol in people with an intermediate level of education. Studies with the laboratory PNS data^5^ showed that high levels of total cholesterol and LDL are less frequent in more educated adults and the prevalence was lower in individuals with average schooling compared with those with a lower level of education.^5^ ^,^ ^11^ The prevalence of lipid profile changes for total cholesterol, according to years of study, were: 37.1% (zero to eight years), 28.6% (nine to 11 years) and 30.4% (over 12 years);^11^ and for LDL: 21.5% (zero to eight years), 16.8% (nine to 11 years) and 16.7% (over 12 years).^5^ ^,^ ^11^ Possible explanations for the occurrence of these findings are: higher demand for healthcare due to a greater understanding of the disease and the inherent risks,^19^ as well as the adoption of further practices in terms of preventive actions, health promotion and healthcare.^11^ ^,^ ^19^ ^,^ ^25^

Regarding race/skin color, the data from this study were similar to population-based investigations in Brazil,^5^ ^,^ ^26^ in which lower prevalence of dyslipidemia was found among those of Black and Brown race/skin color. There is little information on the lipid profile of admixed populations, but it is known that there are differences between ethnicities.^26^ In Blacks, a lower prevalence of high levels of LDL and triglycerides is documented in comparison with Whites; however, people of Brown race/skin color have lipid concentrations close to that of Whites.^26^ Black people have lipid patterns associated with a lower risk of cardiovascular diseases.^26^ Given the lack of information on this topic in admixed populations such as in Brazil, and the differences in the lipid profiles,^26^ further studies are needed to elucidate the potential differences in the country.

Some socioeconomic proxy variables are related to cardiovascular risk factors.^25^ People who use health insurances generally have a higher income, which enables access to services and diagnosis.^23^ Having access to a health insurance may have contributed to the higher number of diagnoses, given that a higher prevalence of health service use was observed, in Brazil, in people with NCDs and who have health insurance plans.^23^ In addition, dyslipidemias are highly prevalent in developing countries, and individuals in higher socioeconomic strata are at increased risk of developing NCDs.^27^ This is due to higher rates of obesity and overweight in such countries,^25^ which contribute to the occurrence of dyslipidemias.^6^ ^,^ ^27^ ^,^ ^28^

The positive associations found between obesity and overweight and a diagnosis of high cholesterol are in line with other studies.^5^ ^,^ ^7^ ^-^ ^9^ ^,^ ^18^ In overweight and obesity, the occurrence of insulin resistance is related to increased cholesterol levels.^28^ This is due to the elevations of free fatty acid concentrations, with increased hepatic secretion of very low density lipoproteins (VLDL), with consequent metabolization of VLDL to LDL particles (small and dense) that accumulate in the vasculature, in addition to elevations of triglycerides.^28^

The NCDs studied herein (hypertension, diabetes and renal failure) are associated with dyslipidemias.^1^ ^,^ ^29^ ^,^ ^30^ In arterial hypertension, atherosclerosis affects the elasticity of the arteries, with increased blood pressure and endothelial dysfunction, increasing vascular permeability to lipoproteins, favoring the accumulation, oxidation and immunogenicity of LDL.^1^ People with renal failure present changes in the lipid profile due to abnormalities in lipoprotein metabolism and, as the renal function deteriorates, triglycerides and LDL concentrations increase and HDL concentrations decrease.^29^ Dyslipidemias can be secondary to diabetes,^1^ mainly due to insulin resistance, resulting in retention of dense LDL particles and the presence of low HDL, which is a common abnormality in people with diabetes.^30^

This study showed a positive association between worse self-rated health status and the diagnosis of high cholesterol. This data is relevant, since self-rated health status is an important predictor of mortality and morbidity.^7^ The findings in this study are consistent with other studies that identified an association between fair, poor or very poor self-rated health and dyslipidemia.^5^ ^,^ ^7^ Possible explanations are the perception of the disease in terms of its consequences and functional changes.^7^

The positive associations between engaging in physical activities and being a former smoker, and the negative association between being a smoker and the diagnosis of high cholesterol may be consequences of changes in lifestyle and treatment. It can also be a possible effect of reverse causality, suggesting that adults with high cholesterol adhered to those changes as a result of the diagnosis. Evidence suggests that physical activity increases HDL, reduces VLDL and triglycerides and increases resistance to LDL oxidation.^1^ Smoking results in endothelial dysfunction and promotes atherosclerosis; smoking causes an increase in total cholesterol and LDL levels, and a decrease in HDL, with smoking cessation being beneficial at any stage of life.^1^ Additionally, the positive association between alcohol abuse and high cholesterol diagnosis, identified in this study, is worrying, since the combination of abusive use of alcohol and saturated fatty acids can increase elevations in triglycerides; thus, reducing the consumption of alcoholic beverages is recommended.^1^

The prevalence of self-reported dyslipidemia was high among adult Brazilians. The factors associated with the diagnosis of high cholesterol were: being female, ageing, higher socioeconomic status, worse self-rated health status, worse self-assessment of health status, having hypertension, diabetes and having renal failure, overweight and obesity, being a former smoker and a smoker, being physically active during leisure time, and being of Black and Brown race/skin color, besides alcohol abuse. This study can provide support in relation to health promotion public policies, the development of clinical protocols in the scope of the Brazilian National Health System, and support for actions to prevent and decrease the rates of dyslipidemia and cardiovascular diseases.

## Supplementary Material 1

**Table:** Variable construction and calculation methods, 2019 National Health Survey, Brazil

Variables	Questions from the 2019 PNS^a^	Calculation method
Self-reported high cholesterol diagnosis^b^	Q60. *Has any medical doctor ever diagnosed you with high cholesterol?* Response options: yes or no.	Number of adults with a self-reported diagnosis of high cholesterol [Q60 = 1] /Number of adults interviewed age ≥ 18 years x100.
Recommenda-tions from health professionals due to high cholesterol^b^	Q62a. *During a consultation for high cholesterol, has a doctor or health care professional ever given you any of these recommendations?* Response options: *Maintaining a healthy diet? Maintaining adequate weight? Doing physical activity regularly? Taking medication? Not smoking? Having regular follow-ups with a health professional?* Yes or no.	Number of adults with a self-reported diagnosis of high cholesterol who received recommendations from a doctor or health professional [Q62a = 1] /Total number of adults age ≥ 18 years who were interviewed and reported a diagnosis of high cholesterol [Q60 = 1] x100.
Mean age at the time of first diagnosis of high cholesterol^c^	Q61. *How old were you when you were first diagnosed with high cholesterol?* Response options: age in years or less than a year.	Mean age at the time of first diagnosis of high cholesterol for the total number of adults age ≥ 18 years who were interviewed and reported a diagnosis of high cholesterol [Q60 = 1].
Self-rated health status^b^	N1. *In general, how do you rate your health?* Response options: Very good; Good; Fair; Bad; Very bad.	Number of adults who self-rated their health status as good/very good [N1 = 1 or 2], fair [N1 = 3] and poor/very poor [N1 = 4 or 5] /Number of adults interviewed age ≥ 18 years x100.
Self-reported diagnosis of hypertension^b^	Q2a. *Has a doctor ever diagnosed you with hypertension (high blood pressure)?* Response options: Yes or no.	Number of adults with a self-reported diagnosis of hypertension [Male: Q2a = 1; Female: Q2a = 1 and Q2b = 2] /Number of adults interviewed age ≥ 18 years x100.
Self-reported diagnosis of diabetes^b^	Q30a. *Has a doctor ever diagnosed you with diabetes?* Response options: Yes or no.	Number of adults with a self-reported diagnosis of diabetes [Male: Q30a = 1; Female: Q30a = 1 and Q30b = 2] /Number of adults interviewed age ≥ 18 years x100.
Self-reported diagnosis of renal failure^b^	Q124. *Has a doctor ever diagnosed you with renal failure?* Response options: Yes or no.	Number of adults with a self-reported diagnosis of renal failure [Q124 = 1] /Number of adults interviewed age ≥ 18 years x100.
Nutritional status^b^	P1a. *Do you know how much you weigh?* Response options: Yes, how much? (kilograms). Don’t know/Can’t remember.	Number of overweight [BMI = 25 to 29 kg/m^2^] or obese adults [BMI ≥ 30 kg/m^2^] /Number of adults interviewed age ≥ 18 years x100. Calculation of numerator: BMI = weight /(height)^2^. Calculate weight in kilos and height in meters. Weight [P1a = 1]/Height [P4a = 1]^2^
	P4a. *Do you know how tall you are?* Response options: Yes, how tall? (centimeters). Don’t know/Can’t remember.	
Smoking^b^	P50. *At present, do you smoke any tobacco products?* Response options: Yes, daily; Yes, less than daily; I don’t smoke at present.	Number of individuals who are former smokers [P50 = 3 and (P52 = 1 or P52 = 2)] or smokers [P50 = 1 or P50 = 2]/Number of individuals interviewed age ≥ 18 years x100.
	P52. *And in the past, have you smoked any tobacco products?* Response options: Yes, daily; Yes, less than daily; Never smoked.	
Recommended consumption of fruits and vegetables^b^	P9a. *How many days per week do you usually eat at least one kind of vegetable (not counting potatoes, cassava or yam) such as lettuce, tomato, kale, carrot, chayote, eggplant, zucchini?* Response options: Number of days; Never or less than once per week.	Number of adults who consumed vegetables or fruit (including juice) at least 25 times per week, having the minimal consumption of five fruits (including juice) and five vegetables per week/Number of individuals interviewed age ≥ 18 years x100. Calculation of numerator: [((P10A x P9A) + (P19 x P18) + P16A) ≥ 25 and ((P10A x P9A) ≥ 5) and (((P19 x P18) + P16A) ≥ 5
	P10a. *In general, do you eat this kind of vegetable?* Response options: once per day (during lunch or dinner); twice per day (during lunch or dinner).	
	P16a. *How many days per week do you usually drink natural fruit juice (containing the frozen fruit pulp)?* Response options: Number of days; Never or less than once per week.	
	P18. *How many days per week do you usually eat fruit?* Response options: Number of days; never or less than once per week.	
	P19. *In general, how many times per day do you eat fruit?* Response options: once per day; twice per day; three times or more per day.	
Consumption of ultra-processed foods^b^	P6b. *Yesterday, did you take or eat?* Response options: *Soft drink? Canned or carton fruit juice or powdered juice? Chocolate drink or flavored yogurt? Packaged snacks or crackers/saltines? Biscuits/cookies, or sandwich cookies or packet cake? Ice cream, chocolate, gelatin, flan or other industrialized desserts? Sausage, bologna or ham? Sliced bread, hot-dog bun or hamburger bun? Margarine, mayonnaise, ketchup or other processed sauces? Instant noodles, packet soup, frozen lasagna or other premade, processed frozen meal?* Response options: Yes or no.	Number of adults that consumed ultra-processed food the day before the research/Number of individuals interviewed age ≥ 18 years x100. Calculation of numerator: Sum of 1 point for each "yes" response from the sub-items of question P6b. Result ranges from 0 to 10. The consumption of five or more groups of ultra-processed food listed in question P6b was considered.
Alcohol abuse^b^	P32a. *In the past thirty days, did you consume five or more drinks containing alcohol on one occasion?* (One alcoholic drink is equivalent to one can of beer, one glass of wine, one dose of liquor, whisky or any other distilled alcoholic beverage). Response options: Yes or no.	Number of adults that engaged in heavy drinking (five or more drinks) on one occasion [P32a = 1]/Number of adults interviewed age ≥ 18 years x100.
Sufficient leisure time physical activity^b^	P35. *How many days per week do you usually (or used to) do physical activities or practice sports?* Response options: Number or days; never or less than once per week.	Numbers of adults that meet the recommended levels of physical activity (150 minutes or more of light/moderate-intensity physical activity or 75 minutes or more of vigorous-intensity physical activity per week) during leisure time/number of adults interviewed age ≥ 18 years x 100. Calculation of numerator: classify the activity as light/moderate or vigorous [P36]. Calculate the duration of the physical activity in minutes [P37]. Calculate the time spent practicing the activity in minutes, per week [P35].
	P36. *What was the physical activity or sport that you practiced most often?* Response options: walking; treadmill walking; running or jogging; treadmill running; bodybuilding; aerobics/spinning/step/jump; water aerobics; localized exercise/pilates, stretching or yoga; swimming; martial arts and combat; cycling or indoor cycling; soccer; basketball; volleyball; tennis; dance classes; others (specify).	
	P37. *Typically, on days when you practice (practiced) physical activities or sports, how long does (did) that activity last?* Response options: hours and minutes.	

a) PNS: National Health Survey; b) The prevalence (%) was calculated for adults age ≥ 18 years; c) The mean age for adults age ≥ 18 years was calculated.
